# Acting and Dancing during the COVID-19 Pandemic as Art Therapy for the Rehabilitation of Children with Behavioural Disorders Living in Socially Disadvantaged Environments

**DOI:** 10.3390/children11040461

**Published:** 2024-04-12

**Authors:** Diana-Lidia Tache-Codreanu, Andrei Tache-Codreanu

**Affiliations:** 1 Neurorehabilitation Research Laboratory, Medical Rehabilitation Department, Colentina Clinical Hospital, 19–21 Stefan cel Mare Street, 020125 Bucharest, Romania; diana.tache@spitalulcolentina.ro; 2 Doctoral School, The National University of Theatre and Film “I.L. Caragiale”, 75–77 Matei Voievod Street, 021452 Bucharest, Romania

**Keywords:** art therapy, catharsis, acting, dancing, children, rehabilitation, behavioural disorders, COVID-19, physical activities

## Abstract

Art therapy is employed in numerous ways in rehabilitation. This study focuses on an art and movement therapy project carried out during the COVID-19 pandemic. Acting and dancing methods were adapted to produce a short musical film series for ten children from disadvantaged social backgrounds displaying nonorganic behavioural disorders. The aim was to acquire novel ways of expression on the part of the participants to release painful emotions in a safe setting using the method of catharsis through acting and dancing, triggering relaxation as a physiological response and improving their attitude. This study retrospectively analyses the changes in the children’s behaviour and their active participation in the project through quantitative and qualitative research. The children improved their attention and self-esteem, their behaviour became less aggressive and impulsive, and half showed increased empathy. The active participation rate during the project was 82%.

## 1. Introduction

Art therapy involves the use of creative processes in order to help increase one’s wellbeing. There are different types of art therapy, including drawing, sculpture, drama (theatre), music, dance, creative writing, and photography [1]. Art therapy is commonly used in rehabilitative settings in order to promote self-expression and relief from negative emotions, and it is believed to be a coherent self-narrative process contributing to meaning making and relief from traumatic stress [2]. It is already well known that art therapy is engaged with considerable results in a variety of ways in the field of rehabilitation. It can be used in many aspects of an individual’s life, according to his personality, including sensory and motor interaction, perception, cognitive, social, emotional, and spiritual issues. Each of the mentioned aspects requires a particular rehabilitation setting [2]. In art therapy, the final concept of the product and its commercial value for public consumption is considered, in general, to be less critical compared to self-expression and the process of making it. Moreover, the products that occur are often private and remain so [1]. Certain art types used as art therapy are able to enhance social consciousness in communities [3] and promote pro-social behaviour [4].

The art therapist subsequently focuses on the features of art therapy that can best serve the rehabilitation process [5]. In order to become an art therapist, one needs to gain experience in at least one of the following fields of knowledge: medicine, psychology, arts, or social services. During rehabilitation, the art therapist must possess visual art skills and specific knowledge needed to introduce various therapeutic elements depending on the subject’s needs. The visual qualities of media art are essential elements in art therapy and they offer alternative strategies in rehabilitation as they integrate visual, sensual, and kinaesthetic experiences [2]. Symbolic speech and metaphors in emotional expression, along with the combination of verbal and nonverbal communication such as singing and dancing, are encouraged in media visual arts as a way of self-expression and to ‘restore’ the self. From a temporal perspective, when traumatic events disrupt the relationship of coherence between the past, present, and future, a coherent auto-narrative personal experience is necessary to pursue in one’s treatment [6]. 

Another impact of art therapy refers to subjects experiencing social stigma and social isolation associated with conditions such as mental impairments. Art therapy’s benefits include a sense of social inclusion for those experiencing stigma from behavioural problems or living in poverty [7,8]. Some arts interventions that use multiple forms of art are more effective in decreasing the mental-health-related stigma on the youth [9]. Performed in groups or managed by a larger team of art therapists, art interventions can contribute to the feeling of social inclusion [10], even for children [1,11]. Art therapy can also improve the personality development of children from socially disadvantaged backgrounds [12]. 

The context of the COVID-19 pandemic affected significantly more children coming from lower socio-economic family backgrounds than children with higher standards of living [13]. Also, as a result of the social isolation imposed by the pandemic, an increase in mental health challenges has surfaced [14]. 

According to the bio-psychosocial theory, genetic predisposition is combined with stress factors in triggering psycho-social problems in children [5]. Several psychosocial environmental factors, such as a lower socioeconomic status, are associated with chronic stress in adolescents [7], which has a significant negative impact on individuals. The physiological mechanism of the stress response is generally modulated by adrenaline and cortisol, which are stress hormones that alarm the body in extreme situations. However, if stress is encountered daily, the body is prevented from returning to homeostasis, and this can lead to anxiety, depression, suicidal ideation, or even to cardiovascular or immune system diseases. Within children and adolescents, social isolation is associated with depression and anxiety, which contribute to a higher concentration of cortisol [8]. Art therapy can reduce cortisol concentration in saliva samples [15], and art interventions such as visual arts, music, dance/movement, and drama are effective in stress management and relaxation therapy [16]. Furthermore, combining multiple types of creative therapies proved that the result can become more effective than using them separately [17]. 

Using music and dance as a form of art and exercise could contribute to vitality or even experiencing joy, improving the subjects’ moods and mitigating depression [18]. Dance movement therapy (DMT) can offer self-awareness and raise confidence; it can also help children modulate their emotions and accept themselves differently [19]. Dance participation creates physiological responses due to the association between exercise and the secretion of endorphins, thus contributing to the enhancement of chemical neurotransmitters [20]. In the case of children, art offers the possibility of enhancing cognitive skills, which triggers brain and mind development and enhances creative abilities. On the other hand, by allowing the release of feelings in a controlled and safe way in therapeutic settings, catharsis provides relief from painful emotions and triggers relaxation or a change of mood as a psychological response [2].

### The Catharsis Model

The catharsis model was employed because it created a safe context for the participants to express their repressed emotions. Aristotle first introduced the process of catharsis in Poetics, although it was not named so [21], but instead defined as a way of ‘purgation’ of the emotions of ‘pity’ and ‘fear’. Through experiencing the Greek tragedy described by Aristotle, the audience could let go of their inner feelings and gain freedom from their own trapped emotions [21], as they empathise with the characters’ tragic destinies on stage and live them as they were their own. This process is known as ‘identification’, which represents a mechanism by which the audience members receive and experience a novel or a film from inside, “as if the events were happening to them” [22].

Going further than Aristotle’s concept of catharsis [23], Freud and Breuer were the first to use it for therapeutic purposes in their work “Studies on Hysteria” in 1895 [24]. They defined catharsis as “the process of reducing or eliminating a problem through bringing it into the conscious and expressing it” [25], and their method is known as the hydraulic model, as the mind is seen as a container which has limited space for emotions, representing the reason why bottled up emotions explode when released through catharsis [21]. There is a second model of catharsis, the ‘unfinished business’ one, in which the repressed emotions are linked to physical exteriorisation, such as the need to hit somebody to release anger [21]. 

The third and final model, as identified by Guinagh, is the conflict one, which merges the best attributes of the first two. ‘Distressful emotions’ represent emotions that are being felt but are not expressed. These represent a midway point between expressive emotions and defences against their expression. Sadness can become a distressful emotion when released through catharsis in crying, for example, occurring when the suppression of sadness, which until then blocked the exteriorisation of this feeling, is being surpassed [21]. Also, our literature research concluded that some art therapists agree that art steps into the unconscious, allowing the subject to express thoughts and feelings that otherwise would be denied or hidden [26,27].

In all three models, catharsis represents an ‘emotional breakdown’ in the subject’s struggle amid expressive emotions and a failed defence in expressing them [21]. This project created a safe artistic context that could help the participants escape their own defence in expressing distressful emotions. 

Art practices like theatre, music, and dance have specialised approaches as art therapy methods [2]. Therefore, theatre has been used as drama therapy, dancing as dance therapy, and singing as music therapy [28]. It was proved that combining multiple creative therapies can make the result more efficacious [15]. Therefore, a project was created to combine all of these three therapies into one to obtain a more complex and efficient healing process [17]. 

The project consisted of making a musical film series of ten short episodes in which children aged between twelve to fourteen and coming from disadvantaged backgrounds acted, danced, and sang with a therapeutical purpose, with the healing process of the project being essential. All of its steps were carefully designed by a team of artists with art therapy experience and supervised by a psychologist. 

The way all these therapies have been combined into a single project and the research study method through quantitative and qualitative evaluations are discussed further. 

## 2. The Project 

During this artistic, therapeutical, and social project, a drama context was created in which ten teens, during their summer holiday, had the opportunity to learn artistic skills and how to express themselves through them. The children had behavioural disorders and lived in socially disadvantaged environments. A ten-episode musical film series was written, directed, and produced especially for them by a professional film director. They acted on screen together with a professional actress who became, together with the entire film crew, an active participant in the therapeutic art process. Professional equipment for film shooting and post-production work was employed for the project. The project was made available afterwards on an online streaming platform, which the authors have chosen not to divulge to protect the participants.

The film set for the project was an art school, where all the main scenes were shot. There were two main sets at this location: a regular school classroom and a particular set for private discussions between the teacher and the children. The production took place in Bucharest, Romania in 2020.

As the project took place during the COVID-19 pandemic, special measures had to be taken to minimise the contamination risk. Therefore, the script and shooting schedule were specially adapted to minimise direct contact between participants without wearing a mask. Luckily, in the end, there was no infection reported during or after the shooting. 

The project’s primary goals were the artistic and therapeutical effects obtained through art therapy in children with behavioural disorders who lived in socially disadvantaged environments. The project also had a social goal: enhancing the community’s social consciousness by the artistic value of the final product obtained and presented to the public. This was an ambitious undertaking due to the number of participants who sometimes acted like a group with particular issues and also because the product of art therapy is rarely for public consumption [1]. 

## 3. Project Aims

### 3.1. The Artistic, Educational, and Therapeutical Aims of This Project

The first aim was for the participants to learn new ways of artistic self-expression, such as acting, singing, and dancing; therefore, they were instructed by specialists in acting, singing, and dancing.

The second aim was to create an artistic context for communication, one that was safe, therapeutic, and ensured the use of appropriate settings for releasing painful emotions and triggering relaxation using the method of catharsis. The film director (who was also the screenwriter of the film), the songwriter, the choreographer, and a professional actress worked to realise this aim. 

### 3.2. The Social Aim of This Project

To create a ten-episode professional film series produced explicitly for the participants to be viewed by the community, draw the general public’s attention to their particular social case, and thus sensitise the audience to this type of activity. The project was made available online. It is essential to mention that the authors have chosen not to divulge the path to where the project has been made available online to protect its participants. In order to accomplish the social aim, an entire film production crew was involved (film director, director of photography, sound engineer, editor, colourist, and sound designer).

After the film was posted online, this case series study focused on the method of catharsis in art therapy with filmmaking used to help children. Thus, this retrospective study used all video-recorded images, photos and noted observations made during the shooting. Its purpose was to evaluate the project’s outcomes objectively and whether it had reached its therapeutic aims. 

The research complies with the World Medical Association’s (WMA’s) Declaration of Helsinki. The participants were treated ethically according to the American Psychological Association code of ethics, and the Colentina Clinical Hospital Ethics Committee approved this study. The research study received no funding.

## 4. Case Series Study

### 4.1. Participants

A group of ten children, identified as five males and five females, aged twelve to fourteen, took part in an art therapy activity. The participants came from socially disadvantaged backgrounds, specifically extreme poverty combined with other social issues such as single-parent families, domestic violence, or illiteracy. These issues were associated with signs and symptoms of behavioural disorders (aggression, defiant behaviour, irritability, prone to inviting altercations, distraction, impulsivity, hyperactivity, low self-esteem, lower than standard frustration threshold, lack of empathy) identified by the psychologist before this project. The children, however, were not receiving any medical treatment. They were enrolled in a regular middle school, and during their summer holiday, they had the opportunity to learn artistic skills and how to express themselves through them. The participants were selected based on the principle of availability (convenience sampling) [29] by a specialised nongovernmental association, and this process was undertaken by the psychologist and his team solely based on their behavioural disorders, and any artistic abilities did not dictate it. The psychologist accompanied the children throughout all stages of the project.

The children’s participation in the project consisted of two main parts. In the first phase, which took one month, the children benefitted from acting, singing and dancing classes with experienced teachers who designed activities specifically for the youth. The development of specific skills had the purpose of healing and was thought of as a beginner’s art-making stage. During this time, the second phase was organised by the film director, who also was the art therapist: a musical video mini-series of ten episodes in which the children had to use the skills acquired in the first stage of the project in order to act, sing, and dance. Each of the ten children that took part in the project had an episode devoted to him or her; this was written in a way that would allow each participant to play a character that would potentially trigger a therapeutic benefit for the actor, as it was already proven that filmmaking could be therapeutic not only for its author but also to the people involved creatively in its artistic components, such as acting [30]. The children were reunited in an art gathering group during rehearsals and shooting. 

The film director went through the usual steps of making a film, a method also used by other filmmakers working as art therapists [10]. The script was written, there was a pre-production action plan (planning all the aspects of the shooting, including the shot list), production (the actual process of filming and working with the actors), post-production (editing, sound mixing, colour grading), and, lastly, its presentation (in this case, uploading it to an online streaming platform). In our case, we adopted a remarkable approach for two steps (writing the script and directing the actors) for the children to experience catharsis and fulfil the project’s therapeutic purpose. 

### 4.2. Writing the Script

The therapeutic mechanism of the script was borrowed from drama therapy, representing “the intentional and systematic use of drama/theatre processes to achieve psychological growth and change. The tools are derived from the theatre; the goals are rooted in psychotherapy” [31]. In this chapter, we will explain how this was used as a starting point, as there are essential differences between drama therapy and our approach that will be discussed. 

The story was a screen adaptation written primarily for this project after a musical stage play, adapted and used in the musical film as a therapeutical context in which the participants could open themselves safely in front of an adult. Therefore, the story and characters that were initially written for the stage went through a process of adaptation for the screen. We will now present the synopsis of the screenplay.

**Figure 1:**
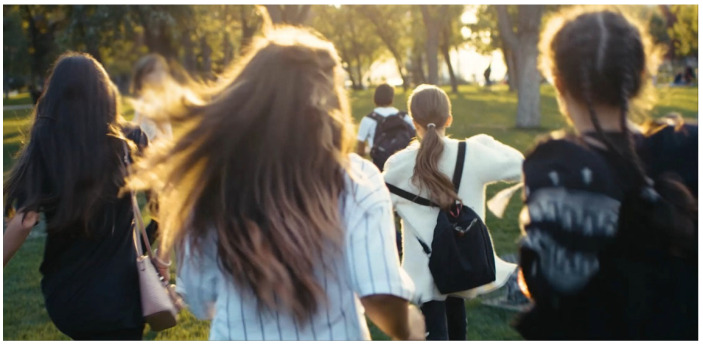
A shot of the children running to the dancing scene.

**Figure 2:**
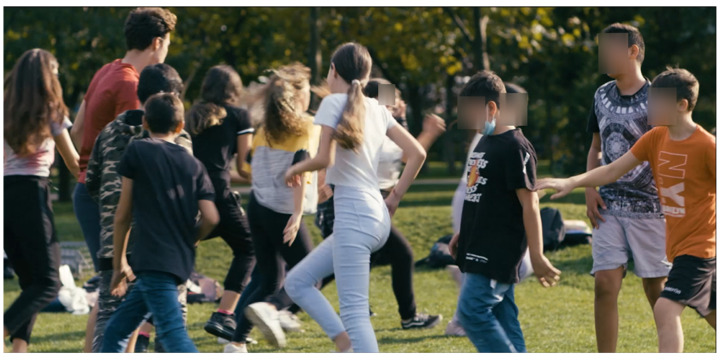
A shot from the film during the dancing scene.

A group of children is discovered by their teacher while fighting with paper rolls during break time. Their disruptive behaviour is punished by the teacher, who is investigating to find out who is guilty of the fight. Each child has a one-to-one discussion with her, explaining the extenuating life circumstances that drive their behaviour and asserting their needs for understanding and validation. At the end of the series, the teacher finally understands her pupils’ problems and needs, and the children celebrate their victory by letting the world know about their problems through a choreographed group song (Figure 1 and Figure 2). As can be noticed, the structure of this story was inspired by that of fairy tales, in which a protagonist encounters the antagonist, engaging in a fight from which the protagonist comes out as a winner, living happily ever after, while the antagonist ends by being defeated and punished. Although the fantastical dimension of fairy tales was not applied here, their structure helped offer the participants a sense of security and satisfaction while participating in the project, knowing they would be winners by the end. The protagonist’s contentment by the end of a story, in the context of discontentment that can be found throughout it, helps inspire hope in children with anxiety. Hope is interpreted as an essential component to living a peaceful and happy life, and the contentment at the end of a fairy tale can help stimulate the faith that all difficulties in life are possible to withstand and still to survive [32]. Although one may observe that fairy tales have a relatively simple literary structure, it comes in contradiction with their possibility to point out complex evolutive, social and individual dynamics, tending to be able to both accommodate and mirror our projections and perceptions [33]. Therefore, the characters were designed in a way that could help the participants think of fairy tales: the characters represented by the protagonists were the virtuous ones fighting in the name of good, and the teacher, the antagonist, portrayed the evil through all her actions, dialogue, and by her way of acting.

All the images presented below are edited or captured from angles that protect the children’s anonymity (Figure 1 and Figure 2).

**Figure 3:**
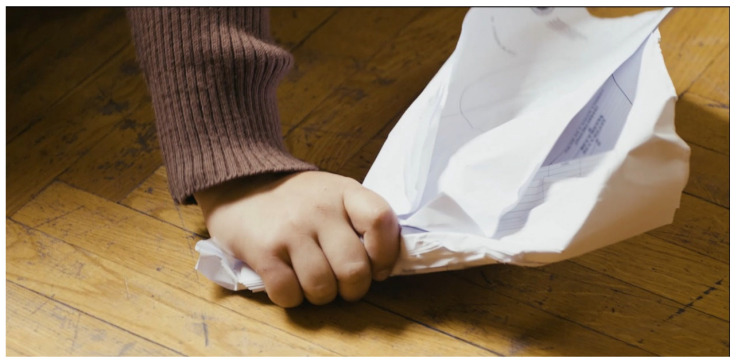
A shot from the film during the paper rolls’ fight.

The characters provide solid arguments stating that the teacher and the other adults from their surroundings are guilty of accusing them of their behaviour. The aim of this story setting was to offer the children a safe medium where they could confront an adult and make him or her understand the characters’ fictionalised problems and inner tensions. Fighting with paper rolls appears to be their way of expressing their problems in the film using familiar channels (Figure 3). 

**Figure 4:**
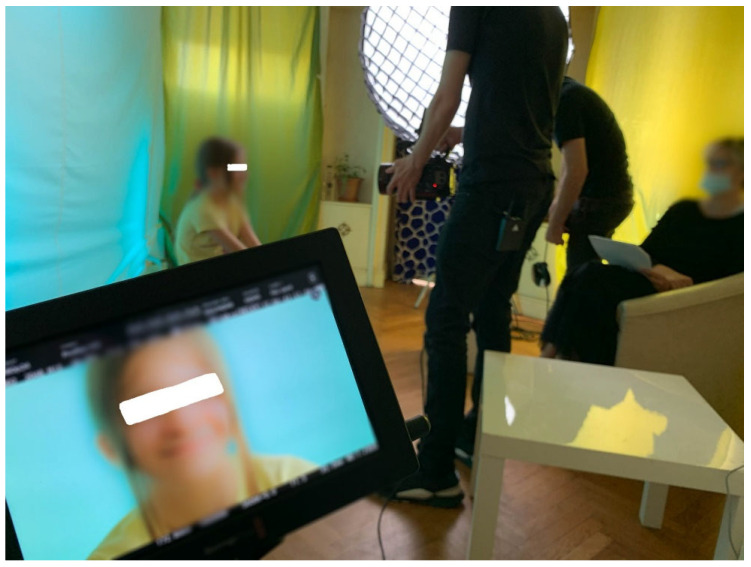
Behind the scenes, the child rehearses in the psychologist’s presence.

The characters’ backstories did not have a direct connection with the participants. These stories were used to offer the children a context in which they could learn how to express themselves, and through the directing process discussed in the next chapter, they were helped to make unconscious connections with their own lives and inner tensions. In this manner, each child could confess to the camera the problems of his or her character openly, unconsciously thinking about his or her personal life. The result was immediate relaxation, the feeling that they had to let go of a heavy burden by expressing themselves in front of an audience and camera crew (Figure 4) while their problems remained a secret.

The most essential aspect from a dramaturgical and therapeutical perspective was each child’s private talk with the teacher. This was a critical moment when each participant switched the discussion from being accused to being the “accuser” of the teacher for not understanding and listening to his or her problems. Therefore, each child came out as a winner from those key scenes, from a dramaturgical point of view for the character played, but most importantly, from his or her perspective. As discussed in more detail later, most of the group showed visible improvement in body posture and attitude after shooting these critical moments. Alongside these dialogue scenes, as in the original musical stage play, dance and singing moments were introduced, in which the children had the chance again to use art to express their feelings. 

The main difference between drama therapy and how this project was scripted is that in drama, the real problems of the participants can be revealed as everything happens in a closed room. In the film, however, it is not recommended, in our opinion, to display the problems the participants faced in their lives. Therefore, finding untraceable roots and links between the child’s personal life and the character played is part and parcel of our methodology. Of course, this step of the process makes it much harder for the art therapist, with the preparation of the therapy being much more complex, yet transferring the classical drama therapy to the film may have, in our opinion, several advantages, such as the following:

- Using professional film techniques, also adapted to be therapeutical, could offer the children a valuable experience in the world of real acting, an experience that they might not otherwise have.
- The presence of the film crew, including a director during the therapeutical process, could help the children feel important and listened to.
- Our work and project presentation create awareness of the social cases presented.

### 4.3. Directing the Actors Using Weston’s ‘Verbs and Facts’ Method Therapeutically 

In this case, the film director worked simultaneously as an art therapist by making a film that was planned to be released to the public and in which the actors played the roles as part of a healing process. Therefore, the techniques of mastering the filmmaking process were adapted to become therapeutical for the children. The method used in this project is described below. 

The central scene in each episode features each child having a personal confrontation with the teacher. These are key scenes for all the episodes not only from a dramaturgical point of view, as they show the children fighting for their rights, but also from a therapeutical perspective, as they represent critical moments for each participant trying to confront the adult in front of him or her. During shooting, Judith Weston’s facts and verbs acting method [34] was adapted for therapeutic purposes. In our project, the film director selected from a variety of methods, specifically these tools, as they had the potential and managed to gain a double role: helping the actors deliver valuable performances on screen and, most importantly, offering them therapeutical relief through how they were employed. 

Verbs were used in the sense that the director would tell the children right before the scene began that their characters were accusing the teacher and that they should say the lines of the script with this particular mindset. By doing so, a feeling of relaxation could be noticed on the part of the children. 

Facts proved to be the most helpful method employed during the shooting. During the aforementioned key scenes involving one-to-one discussions between each child and the teacher, whenever the director noticed that verbs would stop working for a particular child actor, he would guide him or her through facts, such as ‘the teacher in front of you never understood you and your desires’ and ‘she is punishing you all the time without even listening to what you have to say’. The stating of these imaginative backstory facts generated an immediate response, and the child actor would say the lines of the scene in such a truthful way that one could realise that he or she was ‘in the moment’, connected to his personal feelings, a fact considered to be the basis of acting. The production team sensed that these children had been waiting a long time to let go of such emotions in front of an adult [34]. Being ‘in the moment’ directly reflected whether the child experienced visible catharsis while acting, but as it will also be discussed later, the literature proves that catharsis does not necessarily need to be visible in order to exist [32].

Thus, the children were encouraged to relate to the teacher as well as to all the adults that did not seem to understand them in real life. The method was used cathartically, as Freud and Breuer describe it, as each child could express the real unconscious problems of their relationships with adults through their characters. 

The actress playing the teacher was directed to seek an inner evolution during her ten private investigations with all the children. At the beginning of the sessions, she had to be distant and place a barrier between herself and each interviewee, and by the end of the interviews, she had to start empathising with the child and understand him or her. In this manner, the participant would feel understood and listened to, which is an essential factor in the therapy employed in our project. 

The director eased the children’s difficulty in learning the lines by heart, offering them complete freedom to improvise in all situations. This helped them again to have a sense of being ‘in the moment’, resulting in a natural performance on screen and aided their identification with the fictive situations they were playing. 

The dance movement scenes were shot outdoors, in a natural environment, and multiple rehearsals were conducted. The decision to shoot outdoors was determined by the mental health benefits of outdoor exercise [35,36] and the necessity to comply to the safety measures during the COVID-19 pandemic. 

### 4.4. Method of Evaluation

#### 4.4.1. Quantitative Evaluation

In order to achieve a numerical evaluation of the project’s success rate, a series of criteria was employed, for which a score was offered for each child. In order to not become intrusive, direct observation was used for evaluating each case. The team used inter-rater reliability (IRR) measures and noted the honest reactions of the children during the actual shootings and their performances on the screen during the editing phase of the raw materials. It should be noted that this observation method was particularly suitable for this type of project thanks to the possibility of examining the children’s micro-expressions that could be seen during the editing phase in the raw footage. During editing, the footage was analysed carefully from an artistic standpoint to make creative decisions properly during the post-production process. This also helped in making a thorough evaluation of the children’s reactions and behaviour in front of the camera. Moreover, the close-up shots of all the participants, taken by the director during the most important scenes, allowed us to watch everything very closely and analyse their activities, interactions, emotions, and behavioural changes of mood, observed through the lenses of a close camera and video images. Therefore, the camera had a double role: the actual recording of the film and being an “unintrusive witness” to the therapeutical process, capable of capturing reactions which could also be closely studied afterwards and which would otherwise remain unnoticed in a classical direct observation method. 

Therefore, a questionnaire was created based on five items, which were filled out after the shooting for each child. For every item, the Individual Score can be one if the response is positive or zero if the response is negative. In the end, the Total Individual Score can be a maximum of five for each child, as ten children participated in the project. The questions were as follows:

1. Does the child get into character? Does the child improvise in the role within the dialogue scene?
2. Does the child display relaxation during and right after the dialogue scene: facial expression, gesture, body posture (visible catharsis)?
3. Does the child display relaxation during and right after the dance scenes (based on facial expression, gesture, and body posture)?
4. Does the child finalise the given tasks with involvement (acting, dancing, and singing)?
5. Does the child volunteer to help the team with tasks during the film’s production (set decoration, creative input)?

In order to establish inter-rater reliability, the IRR method was chosen and applied to individual and group rating forms for relaxation, improvisation, finalisation rate of the given tasks, and volunteering rate within the project.

Improvising (question 1), completing the project (question 4), and on-set helpfulness (question 5) were objective phenomena, and the inter-rater reliability agreement level was 100%, being observed and discussed by the whole team. Regarding questions 2 and 3, as there are subjective points, the participants’ behaviours were observed in all the shots (the team filmed each child for an average of 4–5 takes to achieve the best result). During the comparison of ratings, even if a team member did not observe relaxation (questions 2 and 3) in a particular take in which the others saw it, the respective member observed it in another take of the same action of the child, also concluding an inter-rater reliability agreement level of 100%. This represents another advantage of this type of film project, as the participants could undergo the same process in different film takes, offering them better chances of experiencing catharsis. The inter-rater reliability rating can also be more precise as different takes could be examined more than once to determine the success of the process.

#### 4.4.2. Qualitative Evaluation

In order to expand and obtain a deeper understanding of the data and the results of quantitative analysis, another evaluation, which will be discussed later, was employed by the film director for the whole group of participants. This analysis was based on direct or noted observations of the children’s behaviour during and between the shooting sessions.

The qualitative research focused on three themes:

- The theme of the analysis of some behavioural changes in the children’s attitude—the subthemes were focused on children’s behaviour observed during the shooting sessions: aggression and inclination to provoke physical aggression, inattention and hyperactivity, isolation and lack of empathy versus social inclusion.
- The theme of children’s treatment compliance—the subthemes were as follows: arrival on time at project sessions, learning the lines, the songs and the choreography, and the children’s motivation in fulfilling all the tasks within the project.
- The theme of post hoc analysis.

## 5. Results

### 5.1. Quantitative Results

The results were added to a total which was used to express the overall percentage of active participation. The five-item questionnaire and the results obtained (Individual Scores, Group Score, and Average Group Score) are presented in Table 1.

For 5 children, the Individual Score was the maximum, being 5 (for participants 1, 2, 4, 6, and 8). This means that half of the participants experienced maximum benefit from the project.

For 3 children, the Individual Score was 4 (for participants 3, 9, and 10) because they all scored 0 on item 5. This means that three children from the total almost obtained maximum benefit from the project.

For 2 participants, the Individual Score was 2 (for children 5 and 7) because they scored 0 on the same items—1, 2 and 5, respectively. This means that only two participants had a low Individual Score, with it being score 2, because they scored 0 on the same 3 items (items 1, 2, and 5), but they recorded a positive response on items 3 and 4. These children experienced a lower benefit from the project. It is essential to mention that no participants had an Individual Score that was lower than 2.

For the first two items of the questionnaire—Item 1: Does the child get into character? Does the child improvise on the role within the dialogue scene? Item 2: Does the child have a relaxation state that can be observed during and right after the dialogue scene, based on facial expression, gesture, body posture (visible catharsis)?—the Group Score (Sum) was 8 (for 8 children, the answer was positive—the Individual Score was 1 for each one; however, for 2 children, the answer was negative—the Individual Score was 0), and the Average Group Score was 80%.

For both the third and fourth items of the questionnaire—Item 3: Does the child have a relaxation state during and right after the dance scenes (based on facial expression, gesture, body posture)? Item 4: Does the child finalise the given tasks with involvement (acting, dancing and singing)?—Group Score (sum) was 10 (for all the children, the answer was positive—the Individual Score was 1 for each child), and the Average Group Score was 100%.

For the fifth item of the questionnaire—Item 5: Does the child volunteer to help the team with any tasks during the production of the film? (set decoration, creative input)—the Group Score (sum) was 5 (for 5 children, the answer was positive—the Individual Score was 1 for each child; however, for 5 children, it was negative—the Individual Score of 0 for each child), and the Average Group Score was 50%.

Based on the results, the Total Group Score (sum) was 41 out of a maximum of 50, and the Total Average Group Score was 82%; hence, the active participation rate at the project was 82%.

### 5.2. Qualitative Results

(a) For the theme of behavioural changes in the children’s attitude from the first shooting session to the last, at the subtheme of aggression and inciting physical aggression, it was noticed that by the end, the attitude of all the children was calmer and more participative with fewer fights and altercations. The only difficulty was posed by the waiting time between shots when the children started to play and discharge their emotions between them in a rather aggressive way. The project psychotherapist supervised and stopped them while the film crew was arranging the lighting. By developing the subtheme of inattention and hyperactivity, it was noticed that the acting lessons and the process of learning the lines and the choreography improved the attention of all the children, helped them concentrate on the acting process, and refrained them from being distracted. Regarding isolation and lack of empathy versus social inclusion, it was noticed that five children interacted with the film crew and helped with the set arrangements. Moreover, one girl actively interacted with the film director, trying to use the clapperboard, and suggested adjusting camera angles to capture a better shot. These children showed empathy towards the film crew, a desire to leave their zone of self-isolation, and a real effort to be socially included.
(b) For the theme of compliance with the art therapy method used, it could be noticed that the children appeared to enjoy the days of shooting. Regarding the subthemes of punctuality and learning the lines and choreography, it was observed that they were very punctual and serious about the activities conducted. Additionally, they were aware that a film shooting involves great effort from a professional film crew, and they followed their role as actors very well.
  With regard to the motivation subtheme, it can be noticed that all the participants were motivated to finish their tasks. As we mentioned above, some girls and one of the boys separated themselves from the rest of the group and tried to help the film crew arrange the set. The group was very involved in the process and felt that we were doing something for their own good, and, in return, they wanted to help the crew, too. This also proved their implication and motivation to participate in the project. As the shooting days ended, the project went through all post-production steps and was finally uploaded to an online streaming platform, where it received approximately 3000 views before this piece was written.
(c) Post Hoc Results: It is worth noting that for the actress playing the teacher, this proved to be a challenging and painful experience—listening to the rather sad stories of each character she interviewed in the film and feeling each child’s emotion. Moreover, because it was challenging to make the children understand that they have to learn their lines prior to the shooting, as professional actors do, the actress that played the role of the teacher actually helped them to learn their parts and went through each line with all the children in the cast. This meant going through approximately twenty-five pages of dialogue. The work was strenuous, primarily due to illiteracy issues in one case and semi-literacy in several other cases. Overall, it would be essential to note that many of the children were involved in all the activities during the shooting sessions, really getting into their characters, improvising the lines, and feeling relaxed during or after the dialogue scenes. As mentioned, some even offered to help the team with practical activities, arranging the set, and showed devotion to the project. Moreover, after the dance scenes, as the director induced a relaxed atmosphere, the children felt encouraged to improvise, enhancing their relaxation and wellbeing.

## 6. Discussions

It could possibly be considered that this type of evaluation could be indirect. However, it has a significant advantage in eliminating children’s dishonest or induced answers. There was an awareness that if the research team asked the children direct questions after finishing the project, they would know that these would have been specially created for them, which may have caused them to provide answers such as “Yes, it has been helpful”. 

As seen in the table, in the first question, eight of ten children offered on-screen authentic performances by which one could feel they were living their character’s life in front of the camera. These eight children managed to go beyond reciting the lines mechanically and started to improvise in front of the camera, living their characters so strong that one could see on the screen a change in their eyes. These children reached a state of relief, relaxation, and wellbeing during and after shooting these scenes, experiencing visible catharsis (question two), an expected outcome that corroborated the data from the literature [2]. As observable behaviour can indicate catharsis, this experience could also occur without the subject having any visible indicators, as some data from the literature suggest [32]. Therefore, there is no proof that the two children who did not express signs of immediate relief did not experience catharsis. However, we will only count the cases with an apparent observable change in their behaviour captured on camera for a more exact measure of the project’s results. We consider that eight clear cases out of ten is a good result as it proves that the majority of the group had a genuine and active involvement in the project and that they reached the expected outcome, experiencing catharsis during the process, through performing the script and performing Weston’s facts and verbs acting method [34] therapeutically. The other two, who did not express a visible catharsis experience during the acting scenes, showed clear signs of feeling relieved and enjoyed the dancing scenes with the rest of the group (question three). In the end, this was a positive and joyful experience for all children involved, and this could be seen in all cases and correlated with the data from the specialised literature [16,18].

Because the score obtained was maximum on question four, we can appreciate that the given tasks were all completed, with good involvement and with the children paying attention to learning the lines, song lyrics, and choreographed dance moves. The fact that they were all able to successfully finish these tasks also helped their self-esteem, an outcome which is in accordance with the current literature [2,19]. 

Five children (question five) volunteered to help the team with different easy tasks during the production of the film series. This proved that they had empathy for the people in the film crew and a desire to become involved and help in the project with practical activities, such as set decorating (the children were not allowed to manipulate heavy or dangerous film equipment that could harm them). This fact showed a desire for social inclusion, stimulated by group art therapies, which is in agreement with the literature [1,10,11]. These five children represented the group that particularly benefitted from the project as they provided positive answers to all five questions. 

The qualitative results were already discussed in some detail as they cannot be separated entirely from the quantitative results due to the numerous correlations that are apparent between them. By the end of the shooting, improvements or positive observations were noticed for all qualitative themes and subthemes. 

By the end, the participants registered behavioural changes in attitude by gaining a calmer and a more participative attitude with fewer fights and altercations, following some of the other literature [37,38]. Attention was improved, and it moved from many other activities to the acting process, with other studies also suggesting that art therapy groups can produce this effect [39]. The empathy and interactions of half of the participants with the film crew proved the desire to leave self-isolation and make visible efforts for social inclusion, as the data from the literature also suggest [1,40]. 

The children were very compliant with this treatment; they showed punctuality, seriousness in learning their part, and motivation for finishing the project. As the shooting days passed, they changed their attitude, which demonstrated confidence and improved self-esteem, a result similar to other art therapy processes [2,19].

It is said that one of the most important differences between “normal” art making and an art therapy process is the final aim. Mastery and control of the artistic process are considered to be either unimportant or much less critical than self-expression, which is considered an essential part of art therapy. Nevertheless, putting one’s feelings and emotions on public display could become therapeutic for individuals, especially for those who see themselves as devalued, incapacitated, or invalid in some way or another [1]. 

A similar project was carried out for war veterans in the United States, in which the participants were responsible for all the creative processes of film creation, from scriptwriting and directing to acting and editing [41]. Although filmmakers assisted them throughout the process, the films had an amateurish look [42]. In our particular case, that of children from disadvantaged backgrounds and environments, acting in a professionally made film aimed to obtain an artistic product to be presented to the public to mitigate their self-devaluation. During the shooting, the participants declared themselves impressed by the professional film crew that worked for their appearance on the screen to be as satisfactory as possible, both from a technical perspective and from an artistic point of view. 

During the dialogue scenes, directing the actors using specific professional acting techniques such as *facts* and *verbs* proved to have a therapeutic effect on eight out of ten of the children as they experienced visible catharsis, resulting in their immediate relaxation and engagement with the scenes, a fact well proven by the camera. This represents an original drama therapy method designed by the film director, acting as an art therapist. 

Although the children did not exhibit artistic skills before the project, they all managed to successfully complete the film shooting through acting, singing, and dancing. This endeavour proved to become more therapeutically efficient by combining multiple types of art therapies, which were correlated with other studies from the literature [17]. Although two of the participants did not experience visible catharsis through acting, they enjoyed the other two processes and successfully finished the project. Integrating the material into a professional short film series was possible, which was another of the project’s objectives. This proved the complete compliance of the children with this complex kind of art therapy used as a rehabilitation method. 

This type of project combines the advantages of individual therapy (focusing on the specific problems of each individual case) with those of group therapy (increasing group communication and the feeling of social inclusion and social interaction, thanks to working directly with the entire film crew).

As a disadvantage, the method involved a relatively high production cost due to the involvement of an entire professional filming and post-production team. Despite this, the effort was worthwhile, and the results are encouraging. A positive development for future similar studies would be introducing a direct evaluation method for the patients for an even more conclusive result. Nevertheless, by asking direct questions, care of planning them must be taken care of in a way that would not make the children feel obligated to provide answers such as ones stating that the project was helpful, as our aim was researching and analysing their honest and unfiltered opinions. This direct evaluation could be made through a series of scales for the psychological assessment of anxiety and possibly depression, or a questionnaire regarding the quality of life before and immediately after the project in order to increase the accuracy of the results, for example, the KIDSCREEN-10 Index [43]; in the medium term, this would also aid in the evaluation of the possible mitigation of anxiety. Also, in the future, for a proper qualitative evaluation, focus group interviews with youth participants could be considered to find out how they felt about the experience in their own words.

Such art therapy activities with media impact help create awareness and empathy for children in need. This could increase the desire of the community to integrate them and focus even more on the social inclusion and social consciousness of these disadvantaged categories of children within the community through similar artistic activities, as another study suggests [3].

As already said above, apart from the chance that the children had to act in a professional production, the videos have gained over 3000 views on an online streaming platform, raising awareness of the children’s situation and of the therapeutic method employed. 

The identical format of each episode that was dedicated for each child offered them an approximative equal time of expression in front of the camera. Moreover, the key scene from a dramaturgical and therapeutical perspective described earlier, which was the private talk with the teacher that each child had in his or her own episode, was shot identically, with all of the children benefiting from a close-up with the same background and the same lighting scheme. This was chosen by the film director in order to build a sense of equality between the fictional characters and also between the children. 

During this retrospective study, scenes that were shot identically were used in order to evaluate the children’s progress during the project. Therefore, bias was able to be avoided in the study thanks to the initial design of the film, which allowed us to analyse all the children within identical conditions. 

The drama therapy method that was used in this pilot project proved to be a valid approach for helping participants with psychological post-traumatic stress to improve their mental health, as another study with a related approach also suggests [44]. Art therapy has also proved to have good potential in improving the behaviour of children in this endeavour, a result that is in agreement with another academic publication [17]. This can be a powerful strategy in mental interventions in vulnerable segments of populations, especially among children [45].

While searching for specialised literature published during the COVID-19 pandemic, from 2020 onwards, focused on projects and studies using art therapy, we were surprised to find very few cases, mainly studying post-traumatic stress among refugees. The present article fills a gap in the sensible field of children from disadvantaged backgrounds with disturbed behaviour, which constitutes an endeavour to use art therapy as an adjuvant through creating a musical film in which children play and dance. There is a need for more applied research in this area to reach definitive conclusions, which could find a practical application in the lives of people from various communities from different parts of the world. After the COVID-19 pandemic, all the parties involved in art therapy (art therapists, students, teachers, and clients) began working together to improve and permanently adapt art therapy as a therapeutic method both in person and online. Moreover, the information presented through this research can be used in situations of discrimination and health inequality, outlining valuable steps for initiating changes [46].

## 7. Strengths and Limitations

After this project and this study came to an end, their strengths and limitations could be observed. 

### 7.1. Strengths and Limitations of This Project

Strengths

- Multiple types of art therapy were combined, contributing towards a more efficient healing process.
- The advantages of both individual and group therapies were able to be combined.
- It facilitated safe social interactions, which were absent during the COVID-19 pandemic.
- The film camera worked as an ‘excuse’ or ‘alibi’, allowing the children to express their feelings openly in front of an adult.
- It included group physical activities in the open air.
- It included learning activities of artistic expression means.
- Awareness of the social cases was presented.

Limitations

- Being a pilot project, an optimum number of participants could not be established beforehand. After finishing this project, it was observed that thanks to the way in which the script was designed, it could have hosted more participants.
- The cost of running this project was relatively high due to the fact that an entire film crew had to be hired.
- The relatively short amount of time the children were involved in the therapeutic activities (approximately one and a half months).
- More resources could have been invested in sharing the episodes with a larger audience than the 3000 viewers that were recorded.

### 7.2. Strengths and Limitations of This Study

Strengths

- Originality—thanks to the novelty of the project.
- As the children were filmed on purpose in close-up shots, during the research period, the team was able to closely and efficiently study their reactions multiple times in the edited and raw footage.
- This study was written retrospectively, and it did not influence the unfolding of our project.
- Qualitative and quantitative methods being used in order to evaluate this project.
- Direct observation was used for the qualitative evaluation.
- As all the children benefited from close-up shots that had an identical look and duration, the team was able to avoid bias in evaluating their progress.

Limitations

- As this study was developed after this project was finalized, the following methods could not be realized for its evaluation: (1) interviews with each child and/or their parents for a quantitative evaluation and according to certain themes and subthemes for a qualitative evaluation; (2) questionnaires/evaluation scales for determining the wellbeing level, anxiety, and depression before and after this project; (3) focus groups in order to discuss based on themes and subthemes for a qualitative evaluation; moreover, we would like to highlight that through a direct evaluation, the risk of the participants feeling obliged to offer positive answers at the end of the project might appear.
- The participants’ benefits could have been observed over a more extended period by introducing an evaluation method made by the psychologist on a medium-/long-term (3/6 months or even one year after the completion of the project) basis in order to observe if the improvements in the children’s behaviour were maintained. In the future, it would be helpful if such a study would be developed in parallel with the project, being able to cover some or even all the above-described limitations.

## 8. Conclusions

Ten children coming from socially disadvantaged backgrounds associated with signs and symptoms of secondary behavioural disorders, yet without suffering from organic mental disorders, acquired artistic skills such as acting, singing, and dancing that they subsequently used in communicating their feelings through appearing in a musical miniseries film. They found a safe setting in this art therapy project specially created for them in order to release painful emotions and to reach a state of relaxation. Both the scriptwriting process and Weston’s ‘verbs and facts’ technique of mastering the film acting process were used in a way that allowed the children to express problems of their characters that were indirectly related to their real lives, proving to have a visible cathartic effect on eight out of the ten participants. We could presume that the remaining two could have also experienced catharsis despite not displaying visible signs of it. Furthermore, including elements of musical filmmaking and encouraging the children to play the characters using dancing and singing techniques, besides the acting ones, offered to all the possibility to express themselves, enhancing the therapeutical effect of this project. While being part of this program, they seemed to have improved their attention and self-esteem, their behaviour became less aggressive and impulsive, and half showed increased empathy towards others.

The final product had a dual role of a film broadcasted on a streaming platform and of a therapeutic process. The awareness of this project was raised thanks to the high quality of the film, which engaged the viewers and sensitised the community to the therapeutic potential of such a process. 

The overall active participation rate of 82% in this project proves, in our opinion, that the findings of this endeavour are promising and might merit replication under more standardised conditions. This project’s successful development in the context of the constraints caused by the COVID-19 pandemic also shows the importance of experiencing social inclusion and outdoor physical activities for healthy behaviour in children.

Although this project had particular strengths, such as combining individual and group therapy methods and using a film camera as an alibi for allowing children to express emotions in front of an adult, it also had certain limitations, such as the high costs and the relatively short amount of time (one and a half months) the children were involved in therapeutic activities. Regarding this retrospective study, which evaluated the outcomes of this project, although the direct observation method proved efficient thanks to the deliberately chosen close-up shots that could be analysed afterwards, it had limitations, such as the lack of questionnaires, focus group evaluations, or a medium-/long-term evaluations.

In conclusion, art therapy proved to be an excellent resource and has great potential for the rehabilitation and behaviour improvement of vulnerable children living in disadvantaged environments. At the same time, there is a need for more applied research in this field to enable it to be practically applied in the lives of various communities from different parts of the world. 

## Figures and Tables

**Table 1:** Quantitative results.

	Questions	Individual Score											
No.		Child 1	Child 2	Child 3	Child 4	Child 5	Child 6	Child 7	Child 8	Child 9	Child 10	Group Score (Sum)	Average Group Score (%)
1	Does the child really get into character? Does the child improvise on the role within the dialogue scene? 1/0	1	1	1	1	0	1	0	1	1	1	8	80%
2	Does the child have a relaxation state that can be observed during and right after the dialogue scene, based on facial expression, gesture, body posture (visible catharsis)? 1/0	1	1	1	1	0	1	0	1	1	1	8	80%
3	Does the child have a relaxation state during and right after the dance scenes (based on facial expression, gesture, body posture)? 1/0	1	1	1	1	1	1	1	1	1	1	10	100%
4	Does the child finalise the given tasks with involvement (acting, dancing, and singing)? 1/0	1	1	1	1	1	1	1	1	1	1	10	100%
5	Does the child volunteer to help the team with any tasks during the production of the film (set decoration, creative input, etc.)? 1/0	1	1	0	1	0	1	0	1	0	0	5	50%
	TOTAL (maximum 5)	5	5	4	5	2	5	2	5	4	4	41 (out of maximum 50)	82%

## Data Availability

The data presented in this study are available upon request from the corresponding author, provided the request is justified, reasonable, and approved by the patient’s representative. Data are not publicly available due to ethical and privacy restrictions because the filmed images contain minors, and their confidentiality needs to be respected.
